# Risk factors for screw loosening in patients with adult degenerative scoliosis: the importance of paraspinal muscle degeneration

**DOI:** 10.1186/s13018-021-02589-x

**Published:** 2021-07-12

**Authors:** Wei Wang, Weishi Li, Zhongqiang Chen

**Affiliations:** 1 Department of Orthopaedics, Peking University Third Hospital, No. 49 North Garden Road, Haidian District, Beijing, 100191 China; 2 Peking University Health Science Center, No. 38 Xueyuan Road, Haidian District, Beijing, 100191 China; 3 Beijing Key Laboratory of Spinal Disease Research, Beijing, China

**Keywords:** Adult degenerative scoliosis, Risk factors, Screw loosening, Paraspinal muscle

## Abstract

**Background:**

Paraspinal muscle is an important component to maintain spinal stability. But the relationship between the degeneration of paraspinal muscle and postoperative screw loosening in patients with adult degenerative scoliosis has not been studied. The objective of this study was to investigate risk factors for screw loosening in patients with adult degenerative scoliosis, including paraspinal muscle degeneration.

**Methods:**

We investigated 93 patients with adult degenerative scoliosis who underwent spinal interbody fusion and pedicle screw fixation surgery. The lateral curvature was located in the lumbar spine and the follow-up time was ≥ 2 years. The patients were divided into loosening and non-loosening groups. Screw loosening was defined as a 1-mm or wider circumferential radiolucent line around the pedicle screw. We checked the cross-sectional area of paraspinal muscles, spinopelvic parameters, bone mineral density, number of fusion segment, and other factors. The potential risk factors for screw loosening were investigated by using binary logistical regression analysis.

**Results:**

Fifty-seven patients showed screw loosening, which is 63.4% of total. Compared with patients in the non-loosening group, the cross-sectional area of erector spinae and psoas major muscle at L5 level were significantly smaller in patients with screw loosening (P < 0.05). Among these factors, the number of fused segments and relative erector spinae total cross-sectional area were independent risk factors for screw loosening.

**Conclusions:**

The degeneration of paraspinal muscle and the increase of fusion segment were independent factors for screw loosening in patients with adult degenerative scoliosis.

## Background

Adult degenerative scoliosis is a common adult spinal deformity, and the prevalence ranged from 30 to 68% in elderly [1–4].These patients often suffer from low back pain, lower limb radiation pain, impaired nerve function, claudication, and so on [5, 6]. For patients without remission under conservative treatment, surgical treatment is required. Screw loosening is a common complication in patients after long-segment fusion and fixation surgery [7, 8], mainly occurring in proximal or distal vertebral bodies, which may be related to osteoporosis and stress concentration after fusion surgery.

Paraspinal muscle is an important component to maintain spinal stability, and the degeneration of paraspinal muscle is associated with a variety of diseases and patient’s outcome [9–11]. In patients with adult spinal deformity, the atrophy of paraspinal muscle was associated with postoperative proximal junctional kyphosis (PJK) [12, 13], but there were few reports on the relationship between the degeneration of paravertebral muscle and screw loosening. A study found that paraspinal muscle degeneration was associated with screw loosening, and smaller cross-sectional area (CSA) and higher fat infiltration (FI) of paraspinal muscle were risk factors for sacral screw loosening [14]. But the study investigated a variety of diseases, including only four patients with adult scoliosis. To the best of our knowledge, the relationship between the degeneration of paraspinal muscle and postoperative screw loosening in patients with adult degenerative scoliosis has not been studied.

The purpose of this study was to investigate the relationship between paraspinal muscle degeneration and postoperative screw loosening and explore the risk factors for screw loosening in patients with adult degenerative scoliosis.

## Methods

It was a single-center retrospective study which was approved by the Ethics Committee of Peking University Third Hospital, and the data of 93 patients with adult degenerative scoliosis who underwent surgical treatment in our hospital from 2010 to 2016 was analyzed. For this type of study, formal consent was not required.

Inclusion criteria were as follows: age ≥ 50 years old, preoperative Cobb angle was ≥ 10°, lateral curvature located in the lumbar spine, the number of fused segments ≥ 3, and the follow-up time was ≥ 2 years. Exclusion criteria were as follows: scoliosis due to other causes, such as congenital scoliosis, idiopathic scoliosis, tuberculosis, and tumors; with a history of spinal surgery; with a lack of complete preoperative and postoperative follow-up data; with application of ilium screw; and so on. All patients underwent posterior median approach without special muscle preservation techniques [1]. The implanted pedicle screws were all conventional screws.

**Fig. 1:**
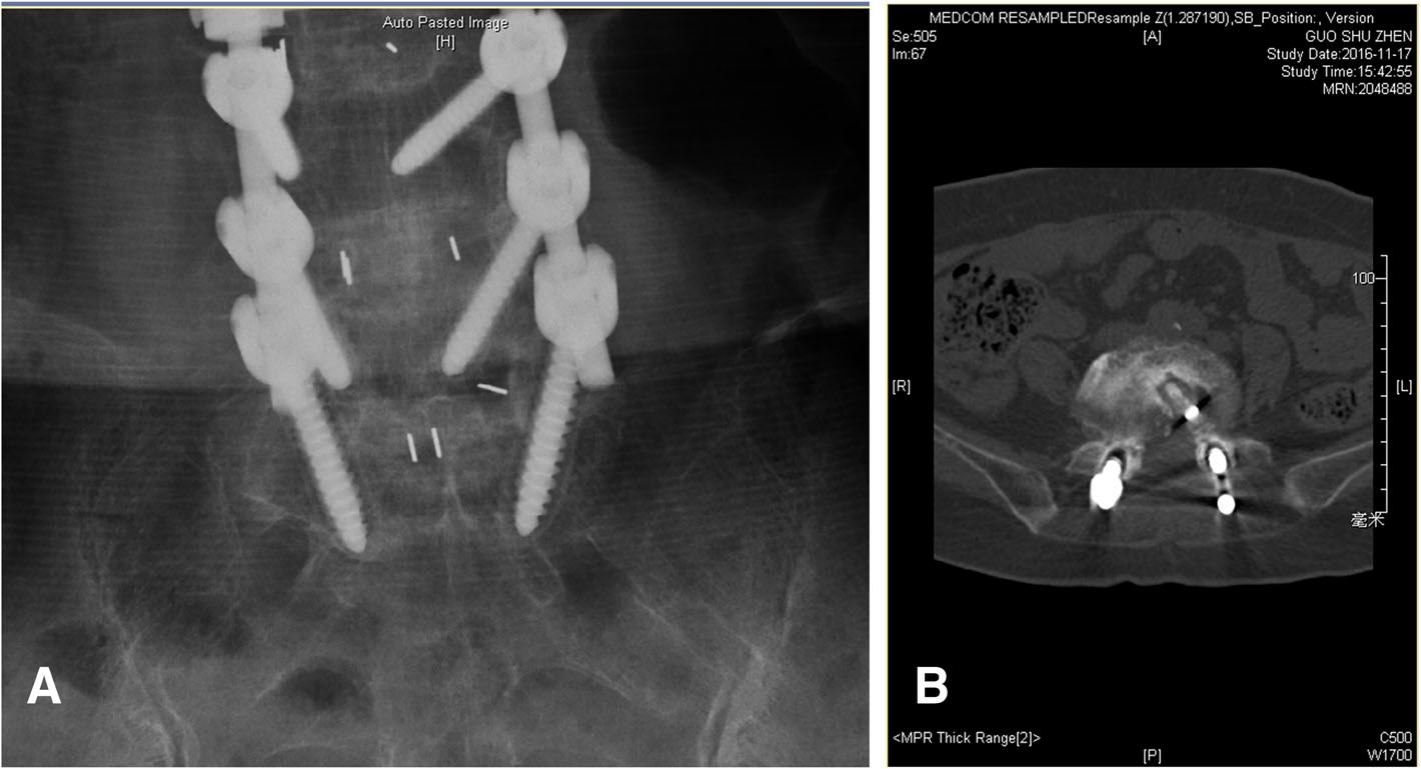
Halo sign around screws on X-ray (**A**) and CT (**B**)

Screw loosening was evaluated by spinal X-ray (Discovery XR650 machine, General Electric Company) and spinal CT (Revolution CT, General Electric Company). As shown in Fig. 1, a halo sign showing a circumferential radiolucent line of ≥ 1 mm around the pedicle screw can be identified as screw loosening [15, 16].

Measurements of paraspinal muscles, including multifidus muscle (MF), erector spinae muscle (ES), and psoas major muscle (PS), were obtained from T2-weighted images. The Signa HDxt 3.0 T machine (General Electric Company) was used for all MRIs, with TR 2500~4000 ms, TE 50~120 ms. The slice thickness was 4 mm with a 4-mm gap between each slice. During the examination, the patient was in the supine position with both lower limbs extended and the spine in neutral position. The plane parallel to the endplate under the corresponding vertebral body (L3-5) was selected for image measurement level.

**Fig. 2:**
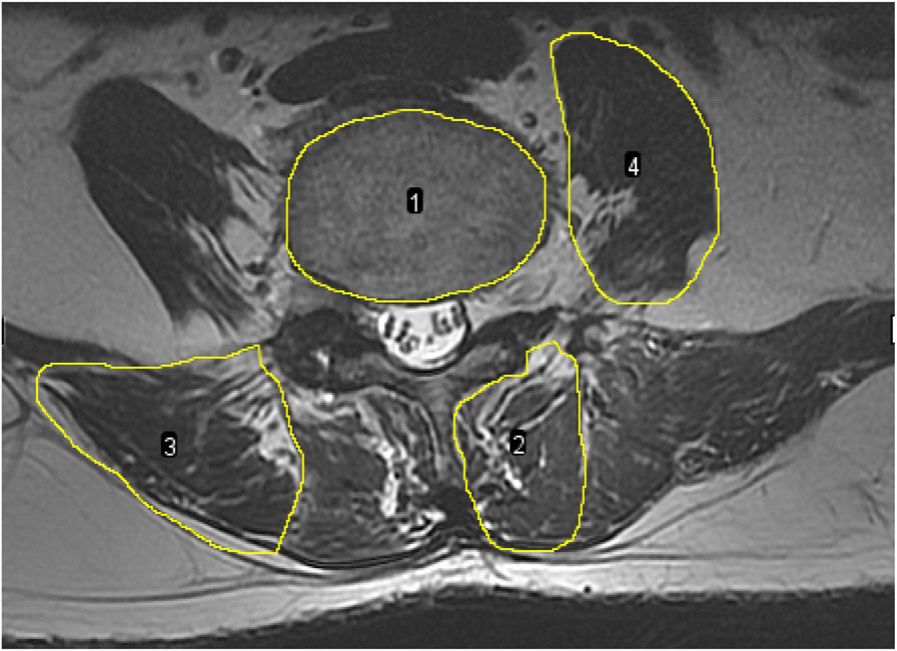
Measurements of paraspinal muscles. (1) Vertebral cross-sectional area; (2) multifidus muscle cross-sectional area; (3) erector spinae muscle cross-sectional area; and (4) psoas muscle cross-sectional area

**Fig. 3:**
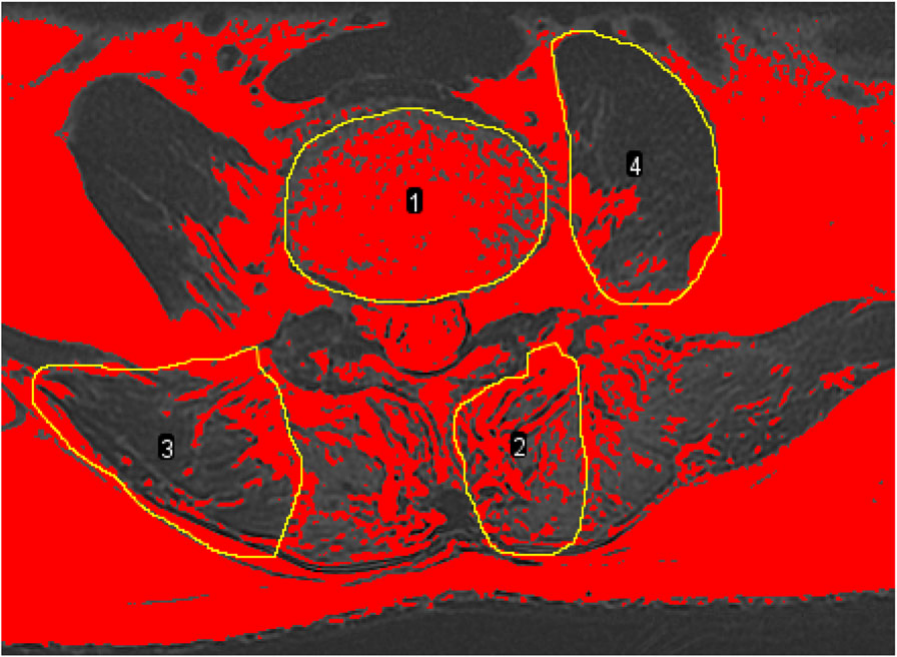
Threshold technique was used to measure the functional cross-sectional area of muscles. Black area stands for the functional cross-sectional area

The paraspinal muscle parameters including vertebral cross-sectional area, muscle total cross-sectional area, and muscle functional cross-sectional area were measured by the Image J software (Fig. 2). The regions of interest (ROI) were outlined around target muscles on the inferior endplate of vertebral body [13, 17]. In order to eliminate the individual differences, the ratio of total muscle cross-sectional area to vertebral cross-sectional area was used as relative total muscle cross-sectional area (rtCSA), the ratio of functional muscle cross-sectional area to vertebral cross-sectional area was used as relative muscle functional cross-sectional area (rfCSA), and the ratio of functional cross-sectional area to the total cross-sectional area (f/t) was used to reflect fatty infiltration (FI). The functional cross-sectional area of the muscle was measured using threshold techniques [17, 18] (Fig. 3).

A Discovery XR650 machine (General Electric Company) was used for all images to measure spinopelvic sagittal parameters The radiographic parameters were measured by standing posteroanterior and lateral whole spine X-ray preoperatively. The parameters including sagittal vertical axis (SVA, the distance between the C7 plumb line and posterior superior corner on the top margin of S1),thoracic kyphosis (TK, the angle between the superior endplate of T4 and the inferior endplate of T12), lumbar lordosis (LL, the angle between the upper endplate of L1 and the sacral plate), pelvic incidence (PI, the angle between a perpendicular from the midpoint of upper endplate of S1 and a line connecting the center of the femoral head to the center of the upper endplate of S1), pelvic tilt (PT, the angle between the vertical and the line through the midpoint of the sacral plate to femoral heads axis), and sacral slope (SS, the angle between the horizontal and the sacral plate) were measured by an experienced orthopedic surgeon.

SPSS version 23.0 (IBM company, USA) was used to analyze the collected data. All values were expressed as mean ± standard deviation. For variables without a normal distribution, the Mann–Whitney U test was used to analyze the difference between different groups, while the independent sample T-test was used to analyze variables with a normal distribution. The potential risk factors for screw loosening were investigated by using binary logistical regression analysis. Statistical significance was set at p-value < 0.05.

## Results

### General data

There were 93 patients in this study, including 21 males and 72 females. The average age of patients was 63.5 ± 5.8 years with a range from 53 to 78 years. The mean follow-up time was 38.4 ± 16.0 (24–48) months. The mean value of body mass index (BMI) was 25.8 ± 4.2 kg/m^2^. The mean blood loss was 1156.6 ± 659.4 ml and the mean operation time was 270.4 ± 61.9 min. The average number of fused segments was 5.5 ± 1.3, including 2 patients with 3 fused segments, 22 patients with 4 fused segments, 28 patients with 5 fused segments, 21 patients with 6 fused segments, 13 patients with 7 fused segments, 4 patients with 8 fused segments, and 3 patients with 9 fused segments. For the distribution of proximal fixed level, 14 patients were at T8-10, 65 patients were at T11-L1, and 14 patients were at L2. For the distribution of distal fixed level, 56 patients were with sacrum fixation and 37 patients were without sacrum fixation. The mean preoperative Cobb angle was 25.9 ± 9.6° and mean preoperative bone density was − 0.9 ± 1.5. For sagittal parameters, the mean SVA was 55.7 ± 38.7 mm, the mean PI was 48.1 ± 10.5°, the mean PT was 23.6 ± 10.3°, the mean SS was 24.5 ± 9.6°, the mean LL was 25.5 ± 15.8°, the mean TK was 21.0 ± 12.3°, and the mean PI-LL was 22.6 ± 16.0° (Table 1).

**Table 1:** Characteristic of patients

Parameters	Value
Age (year)	63.5 ± 5.8
Gender	
Male	21
Female	72
BMI (kg/m^2^)	25.8 ± 4.2
Lumbar T value	− 0.9 ± 1.5
Follow-up time (months)	38.4 ± 16.0
Blood loss (ml)	1156.6 ± 659.4
Operation time (min)	270.4 ± 61.9
Hospital stay (days)	12.6 ± 6.9
Number of fused segments	5.5 ± 1.3
Proximal fixation segment (%)	
T8-10	14 (15.1)
T11-L1	65 (69.9)
L2	14 (15.1)
Distal fixation segment (%)	
Sacrum fixation	56 (60.2)
Non-sacrum fixation	37 (39.8)
Screw loosening (%)	
Loosening	59 (63.4)
Non-loosening	34 (36.6)
Cobb angle (°)	25.9 ± 9.6
Sagittal parameters	
SVA (mm)	55.7 ± 38.7
PI (°)	48.1 ± 10.5
PT (°)	23.6 ± 10.3
SS (°)	24.5 ± 9.6
LL (°)	25.5 ± 15.8
TK (°)	21.0 ± 12.3
PI-LL (°)	22.6 ± 16.0

*BMI* body mass index, *SVA* sagittal vertical axis, *PI* pelvic incidence, *PT* pelvic tilt, *SS* sacral slope, *LL* lumbar lordosis, *TK* thoracic kyphosis, *PI-LL* PI minus LL

### Paraspinal muscle parameters

The measurements of paraspinal muscles, including multifidus muscle (MF), erector spinae muscle (ES), and psoas major muscle (PS), are shown in Table 2. At L3 level, the mean MrtCSA, ErtCSA, and PrtCSA were 80.3 ± 22.5%, 229.8 ± 58.8%, and 95.7 ± 28.8%, respectively. The mean MrfCSA, ErfCSA, and PrfCSA were 41.0 ± 18.4%, 140.6 ± 51.1%, and 87.1 ± 28.1%, respectively. The mean Mf/t, Ef/t, and Pf/t were 49.5 ± 13.3%, 60.5 ± 12.8%, and 90.7 ± 5.8%, respectively. At L4 level, the mean MrtCSA, ErtCSA, and PrtCSA were 109.6 ± 29.0%, 202.3 ± 48.3%, and 129.9 ± 36.9%, respectively. The mean MrfCSA, ErfCSA, and PrfCSA were 55.3 ± 24.0%, 111.9 ± 39.8%, and 119.6 ± 35.0%, respectively. The mean Mf/t, Ef/t, and Pf/t were 49.6 ± 14.0%, 54.8 ± 11.9%, and 92.1 ± 6.2%, respectively. At L5 level, the mean MrtCSA, ErtCSA, and PrtCSA were 135.3 ± 36.3%, 142.4 ± 53.4%, and 135.6 ± 38.2%, respectively. The mean MrfCSA, ErfCSA, and PrfCSA were 62.3 ± 28.1%, 63.0 ± 30.9%, and 133.4 ± 76.2%, respectively. The mean Mf/t, Ef/t, and Pf/t were 45.5 ± 14.2%, 43.7 ± 11.9%, and 97.8 ± 42.2%, respectively.

**Table 2:** Measurements of paraspinal muscles at different levels

Parameters	Value
L3 (%)	
MrtCSA	80.3 ± 22.5
ErtCSA	229.8 ± 58.8
PrtCSA	95.7 ± 28.8
MrfCSA	41.0 ± 18.4
ErfCSA	140.6 ± 51.1
PrfCSA	87.1 ± 28.1
Mf/t	49.5 ± 13.3
Ef/t	60.5 ± 12.8
Pf/t	90.7 ± 5.8
L4 (%)	
MrtCSA	109.6 ± 29.0
ErtCSA	202.3 ± 48.3
PrtCSA	129.9 ± 36.9
MrfCSA	55.3 ± 24.0
ErfCSA	111.9 ± 39.8
PrfCSA	119.6 ± 35.0
Mf/t	49.6 ± 14.0
Ef/t	54.8 ± 11.9
Pf/t	92.1 ± 6.2
L5 (%)	
MrtCSA	135.3 ± 36.3
ErtCSA	142.4 ± 53.4
PrtCSA	135.6 ± 38.2
MrfCSA	62.3 ± 28.1
ErfCSA	63.0 ± 30.9
PrfCSA	133.4 ± 76.2
Mf/t	45.5 ± 14.2
Ef/t	43.7 ± 11.9
Pf/t	97.8 ± 42.2

*M* multifidus muscle, *E* erector spinae muscle, *P* psoas major muscle, *rtCSA* relative total cross-sectional area, *rfCSA* relative functional cross-sectional area, *f/t* the ratio of the functional cross-sectional area and total cross-sectional area

### Comparison between the loosening group and non-loosening group

All patients were divided into the loosening group and non-loosening group according to whether screw loosening occurred in the last follow-up. There were 59 patients in loosening group and 34 patients in non-loosening group. The age of patients with screw loosening was 64.0 ± 6.1 years, while that in non-loosening group was 62.7 ± 5.2 years (P = 0.276). The number of fused segments for patients with screw loosening was 5.7 ± 1.4, while that in non-loosening group was 5.2 ± 1.2 (P = 0.075). Besides, the value of BMI, ratio of gender, bone density, preoperative Cobb angle, and sagittal parameters did not have significant difference between the two groups (Table 3).

**Table 3:** Comparison between the loosening group and non-loosening group

Parameters	Loosening	Non-loosening	P value
Gender (M/F)	16/43	5/29	0.168
Age (year)	64.0 ± 6.1	62.7 ± 5.2	0.276
BMI (kg/m2)	25.5 ± 4.2	26.4 ± 4.2	0.354
Lumbar T value	− 0.8 ± 1.5	− 1.1 ± 1.6	0.434
Cobb angle (°)	27.2 ± 9.2	23.6 ± 9.9	0.078
Number of fused segments	5.7 ± 1.4	5.2 ± 1.2	0.075
Operation time (min)	277.4 ± 66.6	258.4 ± 51.4	0.154
Blood loss (ml)	1142.5 ± 551.8	1180.9 ± 822.2	0.431
Hospital stay (day)	13.1 ± 8.1	11.7 ± 4.1	0.782
Lumbosacral fixation (YES/NO)	37/22	19/15	0.517
Proximal segments (T/TL/L)	10/42/7	4/23/7	0.469
Sagittal parameters			
SVA (mm)	59.4 ± 43.4	49.3 ± 28.5	0.180
PI (°)	47.3 ± 10.0	49.6 ± 11.3	0.308
PT (°)	23.5 ± 10.3	23.9 ± 10.5	0.861
SS (°)	23.8 ± 8.9	25.7 ± 10.8	0.351
LL (°)	24.9 ± 14.8	26.7 ± 17.4	0.604
TK (°)	21.6 ± 13.1	20.0 ± 10.9	0.535
PI-LL (°)	22.4 ± 16.4	22.9 ± 15.6	0.876

*BMI* body mass index, *SVA* sagittal vertical axis, *PI* pelvic incidence, *PT* pelvic tilt, *SS* sacral slope, *LL* lumbar lordosis, *TK* thoracic kyphosis, *PI-LL* PI minus LL

To investigate the correlation between paraspinal muscle degeneration and screw loosening, the measurements of paraspinal muscle were compared between two groups and the results are recorded in Table 4. Compared with patients in the non-loosening group, the ErtCSA, ErfCSA, and PrtCSA at L5 level of patients in the loosening group were significantly smaller (P < 0.05). Therefore, in our study, the atrophy of paraspinal muscle was correlated with screw loosening.

**Table 4:** The difference of paraspinal muscle measurements between the two groups

Measurements	Loosening	Non-loosening	P value
L3 (%)			
MrtCSA	79.7 ± 21.8	81.5 ± 24.0	0.709
ErtCSA	222.6 ± 58.4	242.2 ± 58.1	0.123
PrtCSA	94.0 ± 30.6	98.8 ± 25.6	0.444
MrfCSA	40.8 ± 17.4	41.2 ± 20.4	0.928
ErfCSA	136.0 ± 48.2	148.4 ± 55.8	0.263
PrfCSA	85.7 ± 30.2	89.6 ± 24.1	0.524
Mf/t	50.3 ± 12.3	48.1 ± 14.8	0.445
Ef/t	60.4 ± 12.2	60.6 ± 13.9	0.943
Pf/t	90.7 ± 6.3	90.7 ± 5.0	0.974
L4 (%)			
MrtCSA	108.7 ± 29.4	111.1 ± 28.5	0.706
ErtCSA	199.4 ± 50.4	207.4 ± 44.8	0.443
PrtCSA	129.0 ± 36.6	131.4 ± 38.0	0.756
MrfCSA	54.5 ± 24.1	56.5 ± 24.1	0.700
ErfCSA	110.1 ± 40.3	114.9 ± 39.2	0.581
PrfCSA	118.5 ± 35.8	121.4 ± 33.9	0.698
Mf/t	49.6 ± 14.8	49.6 ± 12.6	1
Ef/t	54.8 ± 11.7	54.9 ± 12.5	0.963
Pf/t	91.7 ± 6.9	92.7 ± 4.6	0.738
L5 (%)			
MrtCSA	131.1 ± 37.2	142.7 ± 34.0	0.140
ErtCSA	130.1 ± 47.4	163.7 ± 57.0	0.003**
PrtCSA	129.3 ± 34.7	146.6 ± 41.9	0.034*
MrfCSA	61.7 ± 27.8	63.5 ± 29.1	0.768
ErfCSA	57.5 ± 27.5	72.6 ± 34.5	0.022*
PrfCSA	132.1 ± 91.9	135.6 ± 36.6	0.130
Mf/t	46.6 ± 14.2	43.5 ± 14.2	0.322
Ef/t	43.9 ± 12.1	43.5 ± 11.6	0.881
Pf/t	100.6 ± 52.8	92.9 ± 4.9	0.116

*M* multifidus muscle, *E* erector spinae muscle, *P* psoas major muscle, *rtCSA* relative total cross-sectional area, *rfCSA* relative functional cross-sectional area, *f/t* the ratio of the functional cross-sectional area and total cross-sectional area

To further explore the effect of paraspinal muscle degeneration on screw loosening, the binary logistic regression analysis was used. The independent variables included age, gender, BMI, number of fused segment, ErtCSA, lumbosacral fusion, and preoperative Cobb angle. As the result shown in Table 5, the number of fused segment and ErtCSA were independent factors for screw loosening. Compared with patients with short fused segments, patients with more than 5 fused segments had a 2.882-fold risk of screw loosening. The greater the ErtCSA was, the lower the rate of screw loosening was. Therefore, in our study, paraspinal muscle plays a protective role in screw loosening.

**Table 5:** The risk factors for screw loosening

Parameters	Regression coefficient	P value	OR value
Fused levels	1.059	0.029	2.882 (1.115–7.454)
ErtCSA	− 1.142	0.013	0.319 (0.130–0.782)
Consistent value	1.792	0.016	

*E*, erector spinae muscle; *rtCSA*, relative total cross-sectional area

## Discussion

Screw loosening is a common complication for patients with fusion and fixation surgery and it is influenced by many factors, such as bone density and operation segment [14, 19, 20]. As an important composition to maintain the stability of spine, the degeneration of paraspinal muscle is of great interest [21, 22]. There was one study found that the degeneration of paraspinal muscle was correlated with S1 screw loosening [14]. But the relationship between the atrophy of paraspinal muscle and screw loosening was unclear in patients with adult degeneration scoliosis.

A 1-mm or wider circumferential radiolucent line around the pedicle screw can be identified as screw loosening [15, 16]. The incidence of screw loosening ranged widely among those studies. Tokuhashi et al. found that the rate of screw loosening after single-segment fusion operation was 7.4%, while the incidence was more than 40% in patients with three or more fused segments [23]. In our investigation, the incidence of screw loosening was 63.4%, which was higher than previous studies. It may be related to the fact that the patients in this study were older and the fused segments were longer compared with previous study [23].

The gender, bone density, and sagittal parameters were not significantly different between the loosening group and non-loosening group. Compared with patients in the non-loosening group, patients in the loosening group were older and their number of fused segments was longer, but their difference was not statistically significant. Among these paraspinal muscle parameters, the CSA of ES at L5 level was significantly different between the two groups. The CSA of ES at L3 and L4 level was not statistically significant between the two groups. By binary logistical regression analysis, we found that the number of fused segments and degeneration of paraspinal muscle were independent risk factors for screw loosening.

The number of fused segments was a common risk factor for screw loosening. With increased fused segments, the incidence of screw loosening increased significantly [14, 23]. Kim et al. found that the rate of S1 screw loosening was higher in patients with 3 or 4 fused segments than 1 fused segment [14]. In this study, all patients had 3 or more fused segments and the rate of screw loosening in patients with more than 5 fused segments was higher than that in patients with 3 to 5 fused segments by almost thrice. The possible explanation was that longer fused segments restricted the motion of spine and increased the stress on screw. So we suggest to consider the fused segment before surgery.

Degeneration of paraspinal muscle was also another risk factor for screw loosening. The cross-sectional area and fatty infiltration of paraspinal muscle were two key parameters to evaluate the atrophy of paraspinal muscle [10, 24–26]. Kim et al. found that screw loosening was related to the degeneration of paraspinal muscle in patients with lumbar degenerative diseases. Patients with screw loosening had smaller CSA of paraspinal muscle at L5-S1 level [14]. But their study only included four patients with degenerative scoliosis. In this investigation, we analyzed the data of patients with adult degenerative scoliosis and found that patients with screw loosening had smaller ErtCSA, ErfCSA, and PrtCSA than patients without screw loosening. By using binary logistic regression analysis, ErtCSA at L5 level was a protective factor for screw loosening.

Paraspinal muscle is important for the stability of spine. Erector spinae muscle and multifidus muscle are important part of extensor muscles to maintain lumbar stability [27–29], while psoas major muscle provides support for flexion of hip joint and lumbar stability [30]. The cross-sectional area of muscle was associated with the muscle strength [31]. Muscle strength decreased with the cross-sectional area decreased, which had a negative influence on the spinal stability and increased the stress of pedicle screw. It may be related to the fact that the rate of screw loosening was higher in patients with worse degeneration of paraspinal muscle.

Fatty infiltration is also another parameter in evaluating the quality of paraspinal muscles. The signal intensity was higher in patients with screw loosening than patients without screw loosening, which suggested that the fatty infiltration was worse [14]. But in this study, the fatty infiltration of paraspinal muscle did not have significant difference between two groups. It may be related to the different measurement techniques. In our study, we measured the fatty infiltration of paraspinal muscle by Image J software, while Kim et al. measured the signal intensity of muscle to reflect the amount of intramuscular fat content [14]. There is not a gold standard in measuring the fatty infiltration of paraspinal muscle, so it needs more efforts to explore this problem. Besides, fatty component in muscles could not provide contractile forces and the fatty infiltration of muscle did not associate with muscle strength [31].

Previous studies reported that the lower bone density and sacrum fusion were risk factors of screw loosening [14, 32]. But in this study, the distribution of both distal and proximal fused level was not statistically significant between the two groups. Another investigation also found that the lumbosacral fusion was not associated with screw loosening [7]. The explanation for different results was that the other factors had a stronger impact than the influence of lumbosacral fixation on pedicle screws stability. Besides, the bone density was not significantly different between the two groups in this study. The decrease of bone density was associated with the increase of age [33]. Patients’ age was not different between two groups, which may lead to the similar bone density.

There were some limitations in this study. Firstly, it was a single-center retrospective study which may bring a selection bias. Besides, the sample size of this research was small. A prospective study with large sample size will be needed to confirm our results. Although it had limitations, this study investigated the relationship between the degeneration of paraspinal muscle and screw loosening. Unlike other studies which only measured paraspinal muscle at one level, we measured the paraspinal muscle from L3 to L5 levels. We found that the decrease of cross-sectional area in erector spinae muscle was independently associated with screw loosening. It could provide help for further prospective investigation about the relation between paraspinal muscles and pedicle screw loosening, the risk factor of screw loosening and so on.

## Conclusions

The cross-sectional area of erector spinae muscle was significantly smaller and the number of operated levels was longer in patients with screw loosening than that in patients without screw loosening. The degeneration of paraspinal muscle and the increase of operated levels were independent factors for screw loosening in patients with adult degenerative scoliosis.

## Data Availability

The data used and analyzed during the current study was available from the corresponding author on reasonable request.

## Abbreviations

BMI: Body mass index
MF: Multifidus muscle
ES: Erector spinae muscles
tCSA: Total cross-sectional area
fCSA: Functional cross-sectional area
FI: Fatty infiltration
PS: Psoas major muscle
